# Nanoscale Extracellular Vesicle Analysis in Alzheimer's Disease Diagnosis and Therapy

**DOI:** 10.1155/2016/8053139

**Published:** 2016-04-26

**Authors:** Pete Heinzelman, Tina Bilousova, Jesus Campagna, Varghese John

**Affiliations:** Drug Discovery Laboratory, Department of Neurology, Easton Center for Alzheimer's Disease Research, University of California, Los Angeles, CA 90095, USA

## Abstract

Diagnostic assays that leverage bloodborne neuron-derived (neuronal) nanoscale extracellular vesicles (nsEVs) as “windows into the brain” can predict incidence of Alzheimer's Disease (AD) many years prior to onset. Beyond diagnostics, bloodborne neuronal nsEVs analysis may have substantial translational impact by revealing mechanisms of AD pathology; such knowledge could enlighten new drug targets and lead to new therapeutic approaches. The potential to establish three-dimensional nsEV analysis methods that characterize highly purified bloodborne nsEV populations in method of enrichment, cell type origin, and protein or RNA abundance dimensions could bring this promise to bear by yielding nsEV “omics” datasets that uncover new AD biomarkers and enable AD therapeutic development. In this review we provide a survey of both the current status of and new developments on the horizon in the field of neuronal nsEV analysis. This survey is supplemented by a discussion of the potential to translate such neuronal nsEV analyses to AD clinical diagnostic applications and drug development.

## 1. Introduction

Bloodborne neuron-derived (neuronal) nanoscale extracellular vesicles (nsEVs) have shown substantial potential as “windows into the brain” that enlighten central nervous system (CNS) disorder-associated changes in brain biochemistry and intercellular communication [1–7]. This review paper describes the current state of neuronal nsEV analysis and also brings to light relatively underexplored opportunities to leverage neuronal nsEV analysis in the context of identifying novel approaches for treating AD. These opportunities may be realized by further developing existing protocols for nsEV isolation to achieve high purity enrichment of bloodborne neuronal nsEVs that enables “omics” profiling of nsEV protein and RNA constituents; such omics profiles could increase our understanding of changes in brain biochemistry and intercellular signaling that both underlie and reflect AD pathology and provide a three-dimensional (3D) nsEV profile. This knowledge may have an important clinical impact by facilitating both identification of novel AD drug targets and development of new molecules and/or modalities for AD prophylaxis and treatment.

## 2. Defining Features of nsEVs

There are no universally accepted criteria for classifying nsEVs. This lack of standard taxonomy creates ambiguity in interpreting and communicating the results of nsEV-related experiments. Our simple classification system defines nsEVs as cell-derived vesicles with submicron diameters and groups them into two categories: exosomes and ectosomes.

Exosomes are manufactured within multivesicular bodies (MVBs), cytoplasmic vesicles that have diameters in the 250–1,000 nm range [8, 9], and are formed by inward budding of late endosomes [10]. Exosome diameters range from 50 to 200 nm [11] and their surfaces are enriched in tetraspanin marker proteins CD9, CD63, and CD81, as well as heat shock proteins such as Hsp70 [12, 13]. Exosomes carry high interior levels of Tsg101 and Alix, two proteins comprising the Endosomal Sorting Complexes Required for Transport (ESCRT) machinery involved intracellular vesicle formation processes [11]. Conversely, ectosomes are vesicles with diameters ranging from 100 nm to 1 micron that bud off from cell plasma membranes.

Others have categorized ectosomes into a number of somewhat ambiguous subclasses: shedding vesicles, microvesicles, exosome-like vesicles, nanoparticles, microparticles, and oncosomes [11]. Although it had been believed that tetraspanin proteins were exosome-specific surface markers more recent analyses have revealed that tetraspanins appear on the surfaces of both exosomes and ectosomes; there are currently no surface marker proteins that distinguish between these two classes of nsEVs [14–16].

nsEVs with diameters between 100 nm and 200 nm can be either exosomes or ectosomes; the vesicle category into which a given nsEV would be classified would be determined by whether the nsEV is formed within a MVB or budded off from the cell plasma membrane. The preceding sentence is framed in a hypothetical sense because, as suggested by the above remarks regarding tetraspanins being common to both exosomes and ectosomes, there are no existing analytical methods that allow one to determine whether a given nsEV isolated from blood was formed inside of a MVB or is instead the product of budding from the plasma membrane.

nsEVs encapsulate nucleic acids, primarily microRNAs (miRNAs) and messenger RNAs (mRNAs). nsEVs also feature integral membrane proteins, proteins covalently bound to nsEV membranes, proteins noncovalently associated with nsEV membranes, and proteins that occupy nsEV interior volumes. nsEVs' principal function is transferring signals from sender to recipient cells. nsEVs originating from sender cells can fuse with membranes of and release their contents into the cytoplasm of recipient cells [17] or have their contents trafficked among different intracellular compartments after recipient cell phagocytosis [18]. nsEV-delivered signals are carried by membrane proteins, interior proteins, miRNAs that suppress transcription of targeted recipient cell genes, or mRNAs that elevate recipient cell translation of the mRNA-encoded proteins.

Given the desire to leverage bloodborne nsEV analysis in enlightening AD-associated changes and brain biochemistry and intercellular signaling processes, we note here that neuronal nsEVs have been observed to migrate from the CNS into the bloodstream in animal experiments [5]. The existence of a similar neuronal nsEV migratory phenomenon in humans is supported by the blood sample AD diagnostic assay results [1–5] discussed in Section 4.

## 3. nsEV Analysis: Current Status and New Developments on the Horizon

The predictive ability of bloodborne neuronal nsEV analysis illustrated by the biomarker validation studies of Goetzl and coworkers, which accurately forecasted AD onset up to ten years prior to clinical diagnosis [1–4], has generated considerable interest in the AD research community. Here we define the state of the art in bloodborne nsEV analysis methods by describing the methods utilized in Goetzl's AD biomarker quantification process. In addition we discuss the potential of 3D nsEV analysis to further build on Goetzl's methods and fully realize the potential impact of nsEV characterization in AD clinical diagnostic platform and drug development.

**Figure 1:**
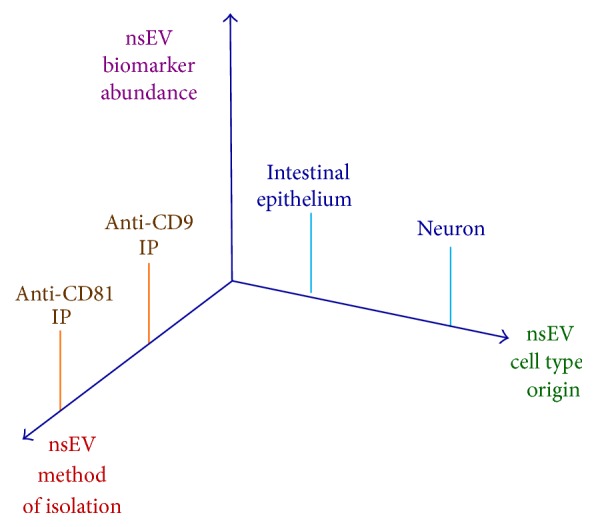
Three-dimensional nsEV phenotyping Cartesian coordinate system. The *x*-axis defines nsEV method of nsEV isolation dimension; anti-CD9 IP and anti-CD81 IP are denoted as two representative *x*-axis points. The *y*-axis defines nsEV cell type origin dimension; intestinal epithelium and neuron are denoted as two representative *y*-axis points. The *z*-axis defines biomarker abundance dimension. The *z*-axis units will vary depending upon the method used to quantify biomarker abundance(s); no representative *z*-axis points are denoted in this figure.

The first step in Goetzl's nsEV AD biomarker quantification process was chemical precipitation (CP) to isolate nsEVs from plasma. After nsEV precipitation, neuronal nsEVs were enriched from the bulk nsEV population using streptavidin-coated agarose beads loaded with biotinylated anti-neuronal marker protein (CD171) Abs. Finally, nsEVs were exposed to lysing conditions and biomarker proteins in nsEV lysates were quantified by ELISA. The Cartesian coordinate system of Figure 1 serves as a useful aid in defining the concept of 3D nsEV analysis; below we provide such a definition by further discussing the steps that comprise the Goetzl biomarker quantification process in the context of this schematic.

As illustrated in Figure 1, each nsEV analysis dimension axis features multiple points that can define a given nsEV characterization experiment. The choice of nsEV enrichment method, that is, CP, size exclusion chromatography (SEC), ultracentrifugation (UC), or immunoprecipitation (IP) [20–23], determines the method of isolation axis coordinate. In the context of Goetzl's works, CP marks the point occupied on the method of isolation axis coordinate. The specificity of the Ab used in IP of nsEVs originating from a cell type of interest, for example, neurons, defines the cell type axis coordinate. In Goetzl's experiments, the use of agarose beads loaded with anti-CD171 Abs for nsEV IP defines the cell type axis coordinate as neuronal.

The third nsEV analysis dimension, biomarker abundance, differs from the other two dimensions in that biomarker abundance, and thus position on the biomarker abundance axis, is dictated by nsEV composition. The researcher chooses which biomarker(s) to quantify and thus defines biomarker abundance axis label(s). As discussed in Section 4, Goetzl and colleagues quantified a set of ten proteins posited to be associated with onset and/or progression of AD. Regardless of whether the researcher quantifies just a few biomarkers, as in Goetzl et al.'s validation studies, or quantifies hundreds of candidate biomarkers in omics analyses, the number of nsEV phenotype coordinate systems will match the number of quantified biomarker species.

## 4. Translational Potential of nsEVs Analyses in AD Diagnosis and Drug Development

The underexplored opportunity in the area of leveraging bloodborne neuronal nsEV omics profiling in AD biomarker discovery for drug molecule and therapeutic modality development is well framed by discussing existing neuronal nsEV AD biomarker validation and discovery-related literature; the latter is particularly relevant to utilizing neuronal nsEV analysis in identification of novel drug targets and establishment of new treatment paradigms. In this discussion we define nsEV biomarkers as protein or nucleic acid nsEV constituents with abundances that differ in diseased relative to normal subjects. This definition encompasses nsEV-associated molecules with relative abundances that change prior to clinical manifestation of disease symptoms and thus can be predictive of disease onset as well as molecules with abundances that change as functions of disease progression or reversion due to positive treatment responses.

We begin our discussion of the literature relevant to nsEV AD biomarker validation by revisiting the works of Goetzl et al. [1–4] and noting that, in addition to accurately forecasting AD onset, Goetzl et al. found that an impressively wide range of proteins, that is, transcription factors [3], molecular chaperones [2], beta-amyloid 1-42 (A*β*_42_) [1], phosphorylated Tau [1], and phosphorylated insulin receptor substrate-1 [4], can serve as predictive AD biomarkers. Also under the umbrella of noteworthy achievements in validating neuronal nsEV constituents as CNS disorder biomarkers, levels of alpha-synuclein (*α*-Syn) carried by bloodborne neuronal nsEVs have been observed to correlate with PD severity [5]. Taken together, the above body of results suggests that bloodborne neuronal nsEV analysis could serve as the basis for clinical diagnostic assays that can predict the onset of and/or monitor the progression or reversion of CNS disorders.

**Figure 2:**
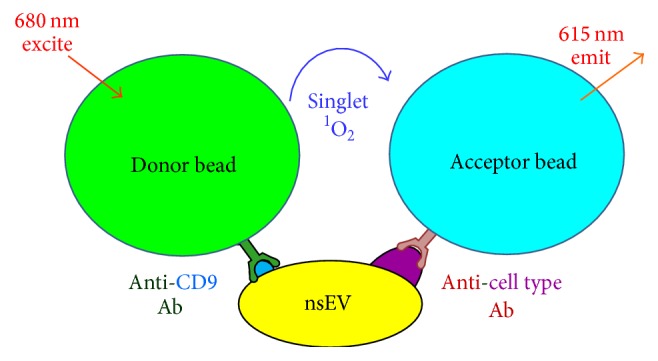
ExoScreen nsEV quantification assay schematic. Upon excitation at 680 nm the phthalocyanine-loaded donor bead converts endogenous diatomic oxygen to singlet oxygen. When donor beads are adequately proximal (~200 nm) to thioxene-loaded acceptor beads donor bead singlet oxygen causes emission of a 615 nm photon from the acceptor beads and produces a luminescent signal detected by the ExoScreen plate reader. For quantification of nsEVs derived from a particular tissue type donor bead exteriors are covalently coupled with anti-CD9 (nsEV surface marker protein) Abs and anti-cell type surface marker protein Abs are coupled to acceptor beads. ExoScreen assays can be carried out in 384-well plates and thus enable parallel quantification of nsEVs derived from multiple cell types in multiple plasma samples. Figure adapted from Yoshioka et al. [19] to illustrate use of ExoScreen nsEV assay for quantification of tissue-specific, for example, neuronal, nsEVs.

**Figure 3:**
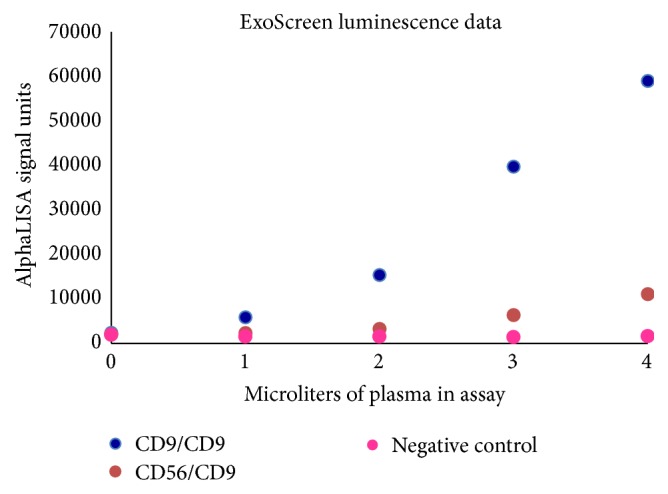
ExoScreen luminescent nsEV quantification assay data for various combinations of AlphaLISA donor and acceptor beads. Data show that ExoScreen allows quantification of neuronal exosomes in plasma. The *y*-axis denotes AlphaLISA luminescence units. The *x*-axis denotes microliters of plasma added to assay. CD9/CD9 donor/acceptor AlphaLISA luminescence data points represent averages of two measurements; values varied by less than 10% across duplicate measurements. Data points for CD56/CD9 and negative control Ab/negative control Ab bead combinations correspond to single measurements.

The bloodborne neuronal nsEV isolation and analysis methods used in Goetzl et al.'s works require more specimen processing steps than are typically associated with clinical diagnostic assays. It is, however, likely that adapting these methods or using other nsEV biomarker measurement techniques could lead to clinically applicable AD diagnostic assays. One such alternative technique is ExoScreen [19], a luminescent AlphaLISA assay-based method for quantifying nsEV biomarkers in blood plasma (Figures 2 and 3). ExoScreen assays are attractive for clinical diagnostic applications as they require only single-digit microliter volumes of blood, are compatible with parallel quantification of multiple biomarkers in multiple plasma samples, and can be completed within just a few hours.

As illustrated in Figure 2, ExoScreen assays can be designed to allow quantification of a biomarker of interest, for example, A*β*_42_, on the surfaces of either the total population of bloodborne nsEVs or only bloodborne neuronal nsEVs; this aspect of the data readout is determined by the Abs that are loaded onto the respective donor and acceptor AlphaLISA beads employed in a given assay. We have observed that ExoScreen can be used to quantify relative levels of total and neuronal nsEVs in plasma (Figure 3). This result suggests that further ExoScreen method development could enable quantification of AD biomarker proteins on the surfaces of bloodborne neuronal nsEVs.

Although considerable progress has been made in validating neuronal nsEV biomarkers for AD diagnostic applications [1–4], much of the script in the arena of discovering neuronal nsEV AD biomarkers for drug target identification and therapeutic development remains unwritten. Such discovery may be enabled by omics methods that characterize neuronal nsEV populations enriched from blood plasma or blood serum. Proteomic approaches, such as tandem mass spectrometry (MS/MS) [24], generate abundance-ranked lists of nsEV-associated proteins present in amounts exceeding proteomic analysis method detection limits, which are generally single-digit picomole. Comparing rank order protein lists for normal and AD subject nsEVs enables identification of new nsEV protein AD biomarkers. Similarly, next generation RNA sequencing techniques, such as Illumina [6], generate global, rank order abundance profiles for bloodborne neuronal nsEV miRNAs and/or mRNAs. Using these profiles to compare levels of RNAs across AD and normal neuronal nsEVs, respectively, could help elucidate novel AD biomarkers.

With respect to the avenues by which omics-based nsEV biomarker discovery can reveal new drug targets and therapeutic approaches for treating AD, we begin by noting that the proteins and nucleic acids contained within nsEVs, as well as proteins both covalently and noncovalently associated with nsEV membranes, reflect the contents of the cytoplasm and/or plasma membrane of nsEV parent cells [11]. As such, AD-associated changes in neuronal molecular composition can be reflected by differences between AD neuronal nsEV composition and the composition of normal subject nsEVs. By extension, correlations between neuronal nsEV composition and the propensity for developing and/or clinically measured severity of AD can inform AD-associated changes in neuronal biochemistry.

These changes in neuronal biochemistry could either play a causative role in AD or be reflective of disease progression. Regardless of which of these cases applies, the knowledge of such AD-associated changes in cellular composition can heighten one's insight regarding how the intracellular molecular milieu is altered either before or after the onset of AD; such augmented insight may add to one's understanding of the mechanisms underlying AD and thus could enlighten novel drug targets or approaches to AD therapy.

An illustration of how the above noted process of using knowledge, obtained via molecular level analysis of bloodborne neuronal nsEVs, regarding AD-associated changes in neuronal molecular composition could be applied in developing new strategies for treating AD can be derived from Goetzl et al.'s studies of levels of ubiquitinylated proteins carried within bloodborne neuronal nsEVs [2]. Goetzl et al. found that ubiquitinylated proteins, where ubiquitin often serves to mark proteins for degradation in lysosomes, were significantly more abundant in bloodborne neuronal nsEVs isolated from AD subjects than in nsEVs isolated from normal persons. This observation implies that ubiquitinylated proteins might be present at elevated levels within the CNS neurons of AD subjects and also suggests that dysfunction of neuronal lysosomes and/or lysosomal trafficking processes could contribute to AD pathology. This posited causative role of dysregulated protein degradation in AD brings modulation of lysosome function and ubiquitinylated protein trafficking within the CNS to light as potential strategies for treating AD.

Neuronal nsEV analysis can also illuminate the bases of AD pathology by virtue of nsEVs' role as facilitators of intercellular communication. Changes in neuronal nsEV signaling molecule levels, particularly RNAs, that correspond to AD can reveal alterations in cell-to-cell communication that are caused by or contribute to AD onset and/or progression. As such, identifying molecules within or on nsEV surfaces with abundances that change in AD subjects facilitates formulation of hypotheses regarding connections between altered intercellular signaling and AD pathology. Knowledge of these differences can help elucidate the mechanisms underlying AD pathology onset and progression; such insights can enable identification of novel drug targets and foster the development of novel AD treatment regimens.

The work of Liu and colleagues [25] provides context regarding how knowledge of AD-associated changes in nsEV signaling molecules levels can enlighten new treatment strategies. Liu et al. observed that levels of miR-193b, which is believed to reduce amyloid precursor protein (APP) expression, are decreased in bloodborne nsEVs isolated from AD subjects relative to normal persons. This finding motivates consideration of drug carrier-encapsulated, CNS-targeted [26] miR-193b as a candidate agent for AD therapy.

The feasibility of proteomic and transcriptomic analyses in bloodborne nsEV CNS disorder biomarker discovery has been established. Tomlinson et al. [24] performed MS/MS analysis on total nsEV populations from serum pools comprised of multiple specimens obtained from respective normal, PD, and amyotrophic lateral sclerosis (ALS) subjects. Eighty-two of the more than 1,000 nsEV proteins in the PD subject pool were absent from the normal subject pool while fifty-four of the PD pool proteins were absent from the ALS pool. Although AD specimens were not included, this work provides precedent for the utility of bloodborne nsEV proteomic analysis in AD biomarker discovery.

Similarly, two independent bloodborne nsEV candidate AD miRNA biomarker discovery efforts have yielded encouraging results. Cheng et al. [6] isolated total nsEVs from sixteen individual AD and thirty-six normal subject serum specimens. Deep sequencing of miRNA extracted from individual specimens identified over 1,400 miRNAs; 16 were either significantly increased or decreased in AD relative to normal nsEVs. This miRNA panel predicted AD with a sensitivity of 77%. Lugli and coworkers [7] performed miRNA deep sequencing of nsEVs isolated from thirty-five normal and AD subject plasma samples, respectively. This analysis identified 465 unique miRNAs; the difference between this number and the more than 1,400 miRNAs reported above results from differences in miRNA abundance thresholds for dataset inclusion. A machine learning algorithm identified seven miRNAs that were differentially expressed in AD subject nsEVs; this panel predicted subject disease status with greater than 80% sensitivity.

Only one miRNA, miR-342-3p, was shared between the above miRNA panels. It is likely that using different nsEV isolation protocols contributed to the lack of overlap between the two predictive miRNA panels. Regardless of these differences, the results of these studies demonstrate the plausibility of using transcriptomics in neuronal nsEV AD biomarker discovery; both these nsEV miRNA studies and the above proteomics study establish foundations for developing 3D neuronal nsEV omics analyses methods that yield highly translatable bloodborne neuronal nsEV protein and RNA profiles.

## 5. Isolation of Bloodborne Neuronal nsEVs

Modifying the total bloodborne nsEV omics analysis protocols used above to enable bloodborne neuronal nsEV profiling for AD biomarker discovery will require resolution of several technical challenges. In this and subsequent sections we identify these challenges and discuss progress to date in and novel approaches for addressing them.

We begin our discussion of these challenges by reviewing bloodborne nsEV isolation procedures. nsEVs can be enriched from plasma or serum using any of the methods enumerated above: SEC, UC, CP, or IP [20–23]. SEC, UC, and CP yield nsEV preparations that contain high levels of contaminants including immunoglobulin Gs, albumin, lipoprotein complexes, protein aggregates or oligomers, and cellular debris [20, 21]. The presence of such contaminants may cause omics analyses to identify false positive neuronal nsEV AD biomarkers by virtue of AD-related differences in protein or miRNA abundance being attributable to the levels of proteins or miRNAs associated with contaminating bloodborne entities rather than those corresponding to neuronal nsEVs. Additionally, if the absolute quantity of a given protein or miRNA carried by bloodborne contaminants is substantially greater than the amount of that same protein or miRNA carried by neuronal nsEVs the resulting high signal-to-background ratio could cause AD-associated differences in neuronal nsEV protein or miRNA abundance to go undetected.

One might find the above concept of a false positive neuronal nsEV AD biomarker to be somewhat paradoxical; if a method for quantifying a plasma AD biomarker accurately differentiates between AD and normal subjects then it would seem that the method is identifying true positives. This apparent paradox is resolved by noting that the “false” adjective applies to the “neuronal nsEV” classifier preceding the “AD biomarker.” If a bloodborne biomarker distinguishes between AD and normal subjects due to abundance differences associated with contaminating bloodborne entities, rather than neuronal nsEVs, then indeed that biomarker is a false positive in the context of accurately reflecting the molecular composition of bloodborne neuronal nsEVs for AD relative to normal subjects. Although false positive nsEV AD biomarkers could be useful for diagnostics applications, their indicating the presence of AD-associated changes in neuronal molecular composition or intercellular signaling processes that may not exist makes them unsuitable as bases for identifying drug targets or developing strategies for AD therapy.

**Figure 4:**
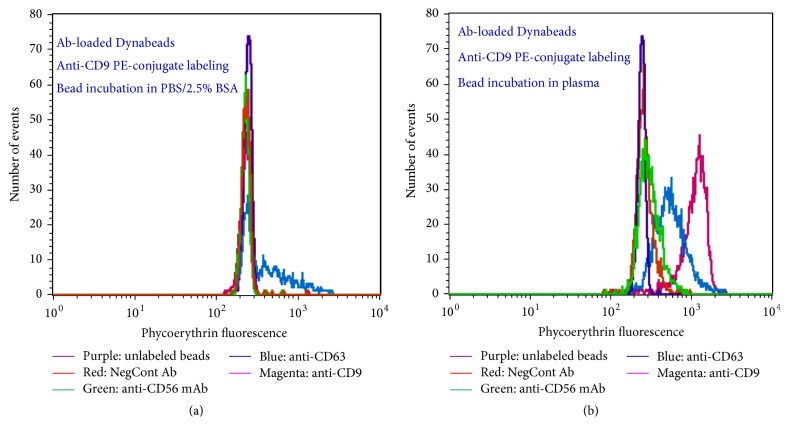
Flow cytometry histograms showing ability to specifically enrich nsEVs from plasma using streptavidin-coated Dynabeads loaded with biotinylated Abs. After plasma incubation, magnetic particles were labeled with phycoerythrin- (PE-) conjugated anti-CD9 Ab. The *y*-axis denotes number of magnetic particle events counted by flow cytometer. The *x*-axis denotes phycoerythrin (PE) fluorescence. Legends under histograms denote biotinylated Abs loaded onto magnetic particles. (a) corresponds to analysis of magnetic particles incubated with PBS/2.5% BSA rather than plasma; these histograms show that PE-conjugated anti-CD9 Ab has little or no propensity to nonspecifically bind Ab-loaded magnetic particles.

We return here to the prior discussion of bloodborne nsEV enrichment methods and note that in seeking to improve upon the purity of nsEV populations isolated using the above noted enrichment procedures we have found that nsEV IP methods employing three-micron diameter magnetic particles (Dynabeads, Life Technologies) loaded with antibodies (Abs) specific for proteins on nsEV surfaces yield high purity nsEV preparations (Figure 4). These IP methods are extremely versatile; streptavidin-coated Dynabeads can be loaded with biotinylated Abs specific for any nsEV surface protein of interest. This versatility, combined with the high purity of enriched nsEV populations, places Dynabeads-based nsEV IP at the heart of our approach for 3D bloodborne neuronal nsEV analysis.

The high purity of Dynabeads-enriched nsEV populations is enabled by an avidity effect arising from respective nsEV and Dynabeads exteriors featuring multiple copies of surface proteins and nsEV surface protein-binding Abs. This avidity effect results in a near-covalent nsEV-Dynabeads interaction that allows one to perform multiple high stringency, for example, pH below 3, wash steps after Dynabeads-plasma incubation without causing nsEV-Dynabeads dissociation. High nsEV purity is obtained by virtue of the wash conditions abrogating the much weaker interactions between Dynabeads and contaminants that are nonspecifically adsorbed to Dynabeads surfaces during incubation with plasma. Dynabeads IP is preceded by moderate centrifugation, for example, 12,000 rcf for 20 minutes, and passage of plasma or serum through a centrifugal or syringe filter with a defined pore size, such as 0.5-micron polyethersulfone (PES). These upstream steps ensure that only plasma constituents presenting nsEV surface proteins of interest and possessing diameters below the filter pore size are enriched during IP. Such Dynabeads enrichment of nsEVs could be useful in the future for 3D nsEV analysis.

PRotein Organic Solvent PRecipitation (PROSPR) is an additional exosome enrichment method that could find future utility in 3D nsEV analysis [27]. The PROSPR method employs a protein precipitation step in which plasma that has been centrifuged to remove whole cells and other large bodies is mixed with ice cold acetone. This precipitation step results in the formation of a solid pellet that contains an appreciable fraction of the albumin and other contaminating proteins present in the starting plasma. After protein precipitation, plasma nsEVs, which remain in the acetone/plasma mixture supernatant, can be exchanged into an aqueous buffer using a centrifugal microconcentrator unit to facilitate subsequent Dynabeads enrichment of tissue-specific plasma nsEV subpopulations.

The PROSPR method has been conclusively shown to yield total plasma nsEV preparations that contain lower levels of plasma protein contaminants than total nsEV populations enriched via UC [27]. In contrast, the EM analyses of nsEV populations enriched using PROSPR that have been reported to date leave ambiguity with respect to what fraction of the enriched nsEVs carry tetraspanin markers [27]. The efficiency of the PROSPR acetone precipitation step in removing cellular debris and large lipoprotein complexes from the plasma supernatant may be lower than the method's efficiency in removing plasma contaminants, such as albumin and immunoglobulin Gs, that are considerably smaller than nsEVs. More conclusively quantifying the fraction of tetraspanin-positive vesicles in PROSPR-isolated nsEV populations, possibly using EM-based approaches similar to those described in Section 7, would provide an increased level of confidence regarding the homogeneity of PROSPR-enriched nsEV populations and thus be a valuable lead-in to employing the PROSPR method in the context of 3D nsEV analysis.

## 6. Two-Step Enrichment of Bloodborne nsEVs for Omics Analyses

Proteomic [5] and transcriptomic [6, 7] bloodborne nsEV CNS disorder biomarker discovery efforts reported to date have employed single-step procedures, for example, UC or IP, to enrich nsEVs for subsequent protein or RNA extraction and omics analysis. Omics analyses that employ the serial Dynabeads IP procedures may increase one's ability to identify neuronal nsEV AD biomarkers relative to omics characterizations based on single-step nsEV enrichment. The low representation of neuronal nsEVs within the total bloodborne nsEV population [1] could cause AD-associated differences in neuronal nsEV constituent abundances to be masked if nonneuronal bloodborne nsEV levels of these constituents are high and invariant across AD and normal subjects. Dynabeads enrichment of nsEVs based on neuronal surface marker proteins, such as CD56 and CD171, may prevent peripheral nsEV (note that CD56- or CD171-based IP enriches nsEVs derived from both peripheral and CNS neurons) and non-nsEV constituents from exerting this masking effect.

Enrichment of neuronal nsEVs for omics analysis requires isolation by IP with Abs recognizing different nsEV surface marker proteins such as anti-CD9, anti-CD63, or anti-CD81. Ab-loaded Dynabeads or ExoCap nsEV IP kits (JSR Micro, Inc.) that include an elution buffer that denatures the nsEV-binding Abs loaded onto the kit's magnetic particles, which are similar to Dynabeads, can be used for the IP. Three-minute incubation of magnetic particles in elution buffer followed by dilution with phosphate buffered saline solution to reduce protein denaturation rate dissociates nsEVs from magnetic particles without causing lysis.

Serial Dynabeads IP can also be facilitated by eluting anti-nsEV Abs from magnetic particles. CELLection Dynabeads surfaces are covalently linked to anti-mouse or anti-biotin Abs by a DNA-based spacer arm. Murine-derived or biotinylated anti-nsEV surface marker protein Abs can be loaded onto CELLection Dynabeads by virtue of associating with the Abs coupled to the CELLection Dynabeads. After plasma incubation and washing, the addition of DNAse-containing reaction buffer releases the Ab-nsEV complexes from the Dynabeads. Ab-nsEV complex elution could also be achieved by using photocleavable linkers [28] to covalently couple Dynabeads to anti-nsEV surface marker protein Abs.

## 7. Quantifying Homogeneity of nsEV Preparations

Carrying out IP of neuronal nsEVs using Dynabeads loaded with anti-neuronal surface marker Abs does not pose elution-associated challenges [5]; bound neuronal nsEVs can be lysed to allow isolation of protein and/or RNA for downstream omics analysis. The isolation of these protein and/or RNA pools should be complemented by estimating the fraction of enriched nsEVs that are neuron-derived; such estimation is needed to ensure that the protein and/or RNA pools being characterized in omics studies are in fact entirely or primarily derived from neuronal nsEVs.

Accurately estimating the fraction of enriched nsEVs that are neuron-derived is a somewhat complex pursuit. Ideal estimation methods would allow one to simultaneously confirm the presence of nsEV and cell type IP surface markers protein on individual nsEVs. Western blot quantification of intraexosomal marker proteins and neuronal surface marker proteins has been used to assess the homogeneity of “neuronal exosome” populations isolated by IP [5] but does not enlighten whether nsEV and neuronal marker proteins are colocalized to the same vesicle; verifying such colocalization requires nsEV imaging.

High nsEV homogeneity with respect to both nsEV and cell type marker proteins is expected for Dynabeads IP. As such, parallel electron microscopy [EM] experiments featuring nanogold-conjugated Ab labeling [5] of respective nsEV and cell type surface markers allow accurate estimation of the percentage of double positive nsEVs. Consider the sample scenario in which 90% of the imaged nsEVs are CD9 (nsEV marker) positive and 75% are CD56 (neuronal marker) positive. In this case between 67.5% and 75% of the isolated nsEVs are double positive. The relative range, that is, upper limit divided by lower limit, of estimated double positive nsEV percentages increases considerably with decreasing nsEV homogeneity. As such, this estimation approach is uniquely enabled by the high purity enrichment techniques such as achieved by Dynabeads IP.

## 8. nsEV Omics Analysis Requirements and Technical Considerations

There are several technical considerations that are important for generating nsEV omics datasets that would enable 3D nsEV analysis such as depicted in Figure 1, as an expansion upon obtaining total plasma nsEV omics data; specimen volume requirements are at the top of this list. Neuronal nsEVs are reported to account for approximately 15% of the total bloodborne nsEV population [1]. Based on this estimate and specimen volumes used in prior omics studies, ensuring adequate neuronal nsEVs for low abundance candidate biomarker detection would require more than 30 mL of plasma for individual specimen proteomic profiling [18] and at least 3 mL of plasma for transcriptomic profiling [6, 7]. The difficulty in obtaining 30 mL of plasma from single subjects will likely necessitate 3D proteomic analyses being performed using pooled specimens. Given that plasma nsEV concentration varies across samples [29], use of nsEV quantification methods, such as NanoSight counting or commercial nsEV surface marker ELISA, could help ensure equal representation of samples comprising specimen pools. We also note that Abs for neuronal nsEV enrichment must be covalently coupled to Dynabeads to avoid dissociation of biotinylated Abs from streptavidin-coated Dynabeads during protein extraction as dissociated Abs contaminate nsEV protein extracts and thus compromise the quality of data obtained during MS/MS omics analysis of nsEV protein constituents.

## 9. Next Generation Tools for nsEV Analysis

Obtaining neuronal nsEV omics datasets using methods described above will provide omics analyses with even greater translational potential. Next generation analyses could be enabled by advances in two emerging areas: microfluidic devices that sort bloodborne neuronal nsEV subpopulations directly from plasma based on abundances of multiple nsEV surface markers and engineered Abs or aptamers that bind epitopes exclusive to AD neuronal nsEV exteriors.

There are two primary obstacles, which arise from nsEV volumes being 10^6^-fold lower than cell volumes, to flow cytometric sorting of individual nsEVs. First, nsEV flow rate must be rigorously controlled to prevent “swarming,” a phenomenon causing groups of nsEVs to be detected as single fluorescence events and leading to encapsulation of multiple nsEVs within sorted fluid droplets [30]. Second, fluorescent Ab-labeled nsEV fluorescence is low relative to the autofluorescence of unlabeled nsEVs and plasma constituents such as cellular debris and protein aggregates. The resulting poor signal-to-noise ratio combines with the effects of swarming to preclude sorting of nsEVs directly from plasma using contemporary flow cytometers; the most advanced plasma-borne nsEV flow cytometry protocols reported to date enable only nsEV analysis [31]. The utility of these cytometry methods is further limited by both their not allowing of detection of nsEVs having diameters of less than 100 nm and their being incompatible with fluorescent Ab labeling of plasma-borne nsEVs; analyzed nsEVs have been characterized based on only size and morphology.

**Figure 5:**
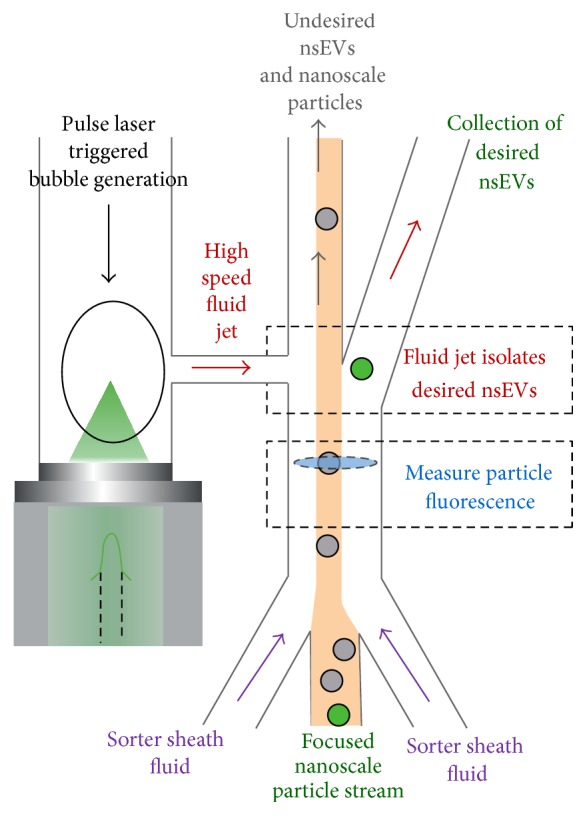
PLACS nsEV sorter schematic. Single nsEVs with desired fluorescence properties, for example, binding by nsEV surface marker- and cell type surface marker-specific fluorescent Abs, are isolated from the bulk plasma nanoscale particle population by high speed fluid jets. These fluid jets are generated by picoliter scale bubbles that are triggered by pulsed laser-induced cavitation. The PLACS sorting module sorts nanoparticles at rates (~30,000/sec) comparable to those for state-of-the-art flow cytometers while providing the advantage of being able to isolate individual nsEVs from the overall particle population. Conventional flow cytometers entrap sorted nsEVs in nanoliter scale liquid droplets; droplets of this size typically encase multiple nsEVs and prevent high purity enrichment of individual nsEVs from the bulk plasma nanoscale particle population.

Further development of existing nonstandard flow cytometers could allow swarming and autofluorescence issues to be overcome. Regarding swarming, Pulse Laser Activated Cell Sorter (PLACS) microfluidics devices [32] achieve exquisite control over particle flow rate and can encapsulate sorted particles in liquid volumes less than 100 picoliters (Figure 5). Time-gated flow cytometers [33, 34], which detect the long-lived photon emissions of lanthanide-conjugated Abs, can improve nsEV detection signal-to-noise ratios by quantifying long-lived lanthanide emissions while filtering out short-lived autofluorescence emissions. Hybrid instruments integrating PLACS fluidics and time-gated optics could be effective apparatuses for sorting individual nsEVs.

Proteins with posttranslational modifications such as glycation, glycosylation, and nitration [35–37] can go undetected or be misidentified by MS/MS proteomic analysis. Additionally, MS/MS proteomics cannot distinguish between different protein conformational isoforms or oligomers. These limitations motivate augmenting panels of candidate AD biomarkers identified using MS/MS proteomics by carrying out nsEV probe (Abs and/or aptamers) library screens to isolate probes binding epitopes unique to or highly enriched on AD bloodborne neuronal nsEVs surfaces. Such probes can be used to immunoprecipitate the proteins carrying AD-specific epitopes for sequence deconvolution [38, 39].

Antigen-loaded Dynabeads are routinely used for probe library screening [40]. As such, performing multiple rounds of alternating negative and positive probe library screens [39, 41] with respective normal and AD bloodborne neuronal nsEV-loaded Dynabeads should yield probes that immunoprecipitate nsEV surface proteins that present AD biomarker epitopes.

Multicolor nsEV cytometry with various combinations of AD nsEV-specific probes and/or commercial Abs against AD nsEV biomarker surface proteins identified by proteomic profiling can address the important question of whether the overall bloodborne neuronal nsEV population is comprised of distinct subpopulations featuring different combinations of AD-specific surface proteins and/or epitopes. Sorting nsEV subpopulations would facilitate downstream omics and/or individual AD biomarker analyses that may enlighten both cell-to-cell heterogeneity of AD-associated changes in neuronal molecular composition and the existence of neuronal nsEVs that carry distinct groups of signaling molecules. Knowledge of such heterogeneity and distinct modes of intercellular communication could enlighten the interplay among different mechanisms of AD pathology and thus be a valuable facilitator of AD drug target identification and therapeutic development.

With respect to forthcoming developments in the area of neuronal nsEV diagnostics hardware for clinical applications, recent progress in miniaturizing complex laboratory operations suggests that the future may prove to hold substantial advances in terms of constructing portable devices for point-of-care quantification of neuronal nsEV biomarkers. Regarding the specifics of this recent progress, protein microarray-based multiplex fluorescent ELISA assays [42] and accurate pinprick volume blood sample HIV diagnosis [43] can both now be carried out using handheld peripheral devices that are compatible with smartphones. Given these impressive achievements, the above envisioned development of portable instruments for quantifying bloodborne neuronal nsEV biomarkers appears to be a realistic goal.

## 10. Conclusion

Advances in neuronal nsEV isolation and analysis methods that leverage neuronal nsEVs could provide “windows into the brain” using noninvasive diagnostic assays, requiring only microliters of blood obtained during routine doctor's office visits, which quantify Alzheimer's Disease (AD) biomarker proteins in nsEVs. Such assays could enable rapid diagnosis for CNS disorders and facilitate the development of personalized treatment programs. Continued increases in public and private funding for nsEV-focused research should enable realization of this goal in a timely manner and hasten the development of next generation nsEV analyses that provide a more accurate analysis of AD biomarkers in plasma. Although it will take time, as has been the case for the human genome sequence, to translate omics-derived AD biomarker discoveries from bench-to-bedside, it is clear that the ever-increasing convergence of biomedical research will augment the relative rate of translation. As such, it is anticipated that we are only years, rather than decades, away from seeing drugs and/or therapeutic modalities inspired by 3D bloodborne neuronal nsEV analyses have a clinical impact in treating this devastating disease.
